# But would you use it again? Determinants of patient intention to reuse and recommend telemental health services: Representative cross-sectional survey from Germany

**DOI:** 10.1177/20552076261450732

**Published:** 2026-07-15

**Authors:** Ariana Neumann, Hans-Helmut König, André Hajek

**Affiliations:** 1 Department of Health Economics and Health Services Research, Hamburg Center for Health Economics, 37734 University Medical Center Hamburg-Eppendorf, Hamburg, Germany

**Keywords:** telepsychiatry, telemedicine, digital mental health, patient adherence, patient preference

## Abstract

**Background:**

To ensure the effectiveness and widespread adoption of telemental health services, continuous use and endorsement by patients must be promoted. Understanding the determinants of patients’ reuse and recommendation of these services is critical, but very limited knowledge exists to date.

**Objective:**

Identifying determinants of reusing and recommending telemental health services among mental health patients.

**Methods:**

Telemental health service users (aged 18-74) who had been receiving mental health services since March 2020 (n=961) were surveyed about their intentions to reuse and recommend the services (0-100% probability scale, continuous variables). A cross-sectional, quota-based (quotas: gender and age group) online survey was conducted in Germany in December 2023. Employing multiple linear regressions, we explored various determinants guided by theoretical models, including socioeconomic, access, health, and psychosocial determinants, as well as patient preferences and provider characteristics.

**Results:**

Higher perceived social support (reuse: *β*=0.37, *p*=.039; recommendation: *β*=0.33, *p*=.044) and more positive attitudes towards telemental health services (reuse: *β*=1.60, *p*<.001; recommendation: *β*=1.47, *p*<.001) were positively associated with the intention to reuse and recommend telemental health services. More positive provider attitudes towards (reuse: *β*=3.35, *p*=.001; recommendation: *β*=3.47, *p*<.001) and greater skills for using the services (reuse: *β*=2.61, *p*=.029; recommendation: *β*=2.68, *p*=.011) were also positively associated with both outcomes. Access, health, and personality determinants showed no significant associations.

**Conclusion:**

Fostering social support and positive attitudes towards telemental health services among patients may enhance the continuous use and recommendation of services. Involving and supporting mental health care providers as advocates for telemental health services may further bridge gaps and promote sustained engagement.

## Introduction

A large proportion of individuals in need of mental health treatment are not being reached effectively by current mental health care systems. The mean treatment gap in mental health care is substantial and ranges from 31% (schizophrenia) to an alarming 76% (alcohol dependence) in the WHO regions.^1^ Major barriers to seeking and staying in mental health treatment are related to low perceived need, as well as attitudinal and structural factors.^2^ Similar to other countries, the German mental health care system is challenged by an overemphasis on inpatient services, poor coordination between inpatient and outpatient sectors, insufficient severity-based treatment allocation, limited adherence to clinical guidelines, and a lack of digitalization and routine outcome evaluations.^3^

Telemental health services, including telecommunications or videoconferencing technology to provide mental health care,^4^ hold great potential to address these issues and have been proven effective among various mental health patient groups (e.g.,^5,6^). Both synchronous (e.g., telephone or video services) and asynchronous (e.g., email exchanges or mobile apps) telemental health services can produce results comparable to in-person mental health services across several outcomes, including clinical effectiveness, diagnostic reliability, working alliance, and patient satisfaction.^5,7,8^ Furthermore, mental health care gaps such as provider shortages, waiting times, stigmatization, or access barriers can be addressed by these services.^9–11^

Nevertheless, remote mental health care faces one great challenge: sustaining real-world patient engagement. Insufficient patient engagement has been repeatedly observed in previous reviews (e.g.,^12–16^), which can restrict the effectiveness of telemental health services (e.g.,^17,18^). For instance, although a recent review and meta-analysis reported a high initial uptake of 92% among mental health app users globally, actual adherence lagged behind at 62%, indicating a gap between initial and continued engagement.^15^ Conversely, studies focusing exclusively on synchronous telemental health services have shown comparable attrition rates to in-person services.^7,19^ Furthermore, a recent meta-analysis has shown that determinants of digital mental health service completion, uptake, and usage differ.^20^ Therefore, engagement outcomes and determinants may vary depending on their measurement and telemental health service type. In contrast to the initial adoption, research identifying the associated factors of continued patient engagement is scarce, yet it is crucial to support the effective use of different modalities of telemental health services in practice.

The Unified Theory of Acceptance and Use of Technology (UTAUT;^21^) emphasizes the role of performance and effort expectancy, social influence, and facilitation conditions, as well as age, gender, experience, and voluntariness of use in shaping the behavioral intention to use telemedicine. Therefore, investigating patient-specific factors and attitudes alongside social and contextual factors is crucial to fully understand the factors associated with sustained service use. In line with the UTAUT, previous studies found associations of female gender, higher treatment expectancy, familiarity with technology, sufficient time, and personalized intervention content, with the willingness to continue using or the continued use of digital mental health services.^22,23^ Besides patient characteristics, recommendations from close others can play a pivotal role in guiding mental health patients towards using telemental health services. Recent studies also suggested a relationship between social influence and the intention to continue using telemedicine.^24–26^ Thus, it may be beneficial to not only enhance reuse but also the recommendation of telemental health services to patients by other patients.

Nevertheless, the existing findings have been generally highly heterogeneous and very limited in scope, often focusing on a narrow range of determinants, services, and sample populations.^19,22,27^ While determinants of use and engagement have been studied to some degree (e.g.,^22,23,28^), no studies have specifically examined factors associated with recommending telemental health services. More broadly, the intention to adopt, patient satisfaction, and service convenience were found to be associated with the intention to recommend or recommending non-mental health-related telemedicine services in single studies with mostly smaller, non-representative samples.^29–31^ Consequently, critical research gaps remain concerning the determinants of reusing and recommending telemental health services, despite their essential role in maintaining engagement with remote mental health care. In particular, studies with larger, representative samples, those that consider different telemedicine service types as well as a wide range of determinants, and research specific to the German mental health care context are needed. Regarding service recommendations, first studies on the determinants of recommending telemental health services are urgently needed, and additional determinants should be explored within larger samples, and for different service types.

### Objective and rationale

Therefore, we aim to identify determinants of reusing and recommending different types of telemental health services (video, telephone, and asynchronous services). This knowledge can assist in fostering continuous engagement of patients with telemental health services. The findings regarding determinants of patient reuse could help to identify target groups who are especially suitable for remote treatment over a longer period, patients who need additional support to prevent discontinuation of treatment, as well as facilitators and barriers to continued use of telemental health services, which could have important implications for research, practice, and policy. In addition, identifying determinants of recommending telemental health services may aid in enhancing social support, which encourages adoption and continued use of the services. Finally, willingness to reuse and recommend health services are established indicators of perceived health service quality and patient satisfaction (e.g.,^32,33^), which may inform telemedicine care practice.

We want to consider a broad range of determinants belonging to various categories. The selection of determinants will be primarily guided by the UTAUT,^21^ given that it is a comprehensive and well-established framework for understanding telemedicine adoption. To attain a more nuanced and thorough understanding of the factors associated with the reuse and recommendation of telemental health services, we will integrate this framework with additional determinants identified through the recent literature that are not explicitly addressed within the original UTAUT model.

Firstly, this will include *socioeconomic determinants* (e.g., female sex, younger age, full-time employment), which have been highlighted as determinants of use or continued use of telemental health services in the UTAUT and past studies.^21–23,28,34^

Second, *access determinants* such as insurance type or internet connection quality at home can play a key role in enabling the continued and satisfactory use of remote mental health services.^35^

Third, *health determinants* may determine suitability (e.g., severity of psychological symptoms^28^) or need (e.g., mobility restrictions^11^) for the continuance of remote mental health treatment.

Additionally, *psychosocial determinants* have rarely been studied, despite the UTAUT and previous research indicating their association with patient engagement in telemental health services, including factors such as social influences, personality, and loneliness.^21,34^

Furthermore, meeting *patient preferences* is crucial for promoting adherence in telemedicine contexts, particularly as outlined in the UTAUT (e.g., effort and performance expectancy). While this factor appears essential in general mental health care,^36,37^ it may be equally important in the context of telemental health care.^34,38^

Finally, *provider characteristics* have the potential to be essential facilitators of the continued use of telemental health services. While research in this area is limited, existing studies highlight providers as gatekeepers and pivotal agents in supporting the use of and satisfaction with remote mental health care among patients.^34,39^ By combining the robust theoretical foundation of the UTAUT with these considerations, our extended framework aims to provide a more comprehensive and context-sensitive analysis of the factors associated with telemental health service reuse and recommendation.

## Methods

### Sample

Our sample included individuals from the general adult population in Germany (aged 18-74), who had been using mental health services since March 2020. To support the representativeness of our sample, the recruitment was quota-based for sex and age groups, established from mental health care utilization rates in Germany.^40,41^ The final sample consisted of 2,178 individuals. For the present study, we exclusively focused on individuals with experience in using telemental health services (n=961). After excluding participants with incomplete data on any of the included predictors (n=27), the final analytic sample included 926 individuals. An a priori power analysis using G*Power (version 3.1) indicated that a minimum sample size of N=178 would be required to detect a medium effect size (f^2^=0.15 with alpha=0.05 and power=0.80), which was largely exceeded in our study. The data collection was realized in cooperation with the market research company Bilendi (ISO 20252:2019-certified online panel provider). Bilendi uses different channels to recruit panel members, including online ads, social media, email, and referrals from other members. The cross-sectional data were collected between December 1 and December 15, 2023, using an online survey (completion rate 89.4%). More detailed information on the online survey is provided in Appendix 1 (Checklist for Reporting Results of Internet E-Surveys). Prior to that, the survey was pretested in November 2023 (n=13), after which minor changes were incorporated to increase clarity and accuracy of the survey. All of the instruments included in the online survey are publicly available and permitted for use in academic research. A few single items were slightly modified to better suit the study context by specifying particular telemental health modalities, such as video, telephone or asynchronous services.

### Ethical considerations

On the first page of the online survey, participants received information regarding the study, including the study purpose, survey contents, data handling, and the responsible investigator. Written informed consent was provided by all panel members by agreement in the online consent form at the beginning of the survey, which is a standard procedure in online surveys. Panel members were able to opt out of the panel at any time. A pseudonymized data set was provided by Bilendi. Participation in the survey was compensated by Bilendi (real-time cash payments between 0.90€ and 1.80€). The Local Psychological Ethics Committee of the University Medical Center Hamburg-Eppendorf (LPEK-0683) approved our study.

### Dependent variables

Our study focused on two main outcomes (see Appendix 4 for overview of included scales and measurement of variables). First, we investigated the mental health patients’ intention to reuse telemental health services using one item. Participants were asked whether they would use telemental health services as part of their mental health treatment again in the future. Second, an additional item examined the intention to recommend the services and inquired whether participants would recommend telemental health services for mental health care. Similar to other large health panel surveys assessing patient expectations (e.g., Health and Retirement Survey,^42^ The Survey of Health, Ageing and Retirement in Europe^43^), the response format included a probability slider ranging from 0 to 100% for both outcomes, allowing for a more fine-grained assessment of patient intentions. Previous research indicates that slider responses are broadly comparable to traditional Likert scales in terms of reliability, validity, and overall data quality, while offering potential advantages such as reduced ceiling and floor effects and enhanced engagement for respondents.^44–46^

### Independent variables

We included several independent variables, based on observed relationships in previous research and theoretical considerations (see Appendix 4 for overview of included scales and measurement of variables).^19,21–23,27^ To test associations of socioeconomic determinants with the outcomes, we included sex (male, female), age (in years), educational level (International Standard Classification of Education 1997^47^: low [ISCED level 0-2], medium [ISCED level 3-4], high [ISCED level 5-6]), employment status (unemployed, full-time employed, part-time employed, other), migration background (yes, no), area lived in (urban, mostly urban, rural), and relationship status (in a relationship, single). Similar to other large surveys (e.g.,^48,49^), the monthly household net income was assessed using 13 different income categories (1 = less than €500, 2 = €500 to under €1000, 3 = €1000 to under €1500, 4 = €1500 to under €2000, 5 = €2000 to under €2500, 6 = €2500 to under €3000, 7 = €3000 to under €3500, 8 = €3500 to under €4000, 9 = €4000 to under €4500, 10 = €4500 to under €5000, 11 = €5000 to under €6000, 12 = €6000 to under €8000, 13 = €8000 or higher; conversion rate in 2023: US $1 = €0.924). For our analyses, these income categories were grouped as terciles (low, medium, high).

Access disparities were considered in our study, including health insurance status (statutory health insurance, private health insurance) and the quality of the internet connection at the patients’ home (fast and stable; fast, but not stable; stable, but not fast; neither fast nor stable, or no internet connection at home).

Regarding health determinants, we measured the presence and severity of depressive (Patient Health Questionnaire-9, PHQ-9^50^) and anxiety (Generalized Anxiety Disorder Scale-7, GAD-7^51^) symptoms in the last two weeks, using well-established and psychometrically sound questionnaires,^52,53^ which also showed good or excellent internal consistency in our sample (Table A1 in Appendix 2). The PHQ-9 comprises 9 items with sum scores ranging from 0 to 27 (higher values reflect more severe depressive symptoms), and the GAD-7 consists of 7 items with sum scores ranging between 0 and 21 (higher values reflect more severe anxiety symptoms). In addition, we assessed the presence of at least one chronic physical illness (yes, no) and self-rated health (5-point Likert scale ranging from 1 = very bad to 5 = very good).

Furthermore, we investigated psychosocial determinants using well-established questionnaires with good psychometric properties.^54–57^ The respective internal consistency values of the included scales are presented in Table A1 (Appendix 2). We measured loneliness using the 6-item De Jong Gierveld Loneliness Scale^55^ (mean scores range between 0 and 11, higher values reflect higher levels of loneliness) as well as perceived social support by family and friends using the 6-item Lubben Social Network Scale^57^ (sum scores range between 0 and 30, higher values reflect greater perceived social support). Moreover, general self-efficacy was assessed using the 3-item Short Scale for Measuring General Self-efficacy Beliefs (Allgemeine Selbstwirksamkeit Kurzskala,^54^ mean scores range between 1 and 5; higher values reflect higher general self-efficacy). The 5-item Satisfaction with Life Scale^58^ was used to examine life satisfaction (sum scores range between 5 and 35, higher values reflect higher life satisfaction). We also measured the patients’ personality using the 15-item Big Five Inventory–Socio-Economic Panel,^59^ which includes 15 items assessing conscientiousness, extraversion, openness, agreeableness, and neuroticism, each with 3 items (trait sum scores range between 3 and 21, higher values indicate higher levels of the different personality traits).

We also considered patient preferences, which included the patients' attitude towards telemental health services. This was measured using the 14-item Unified Theory of Acceptance and Use of Technology Patient-Version Questionnaire.^60^ This scale is based on the widely established framework of the UTAUT^21^ and assesses the patients’ therapy quality expectancy, convenience, ease of use, and pressure from others (sum scores range between 14 and 70, higher values reflect more positive attitudes). A German translation of the scale was developed for our survey with support from two specialized translators from the professional translation agency tolingo (ISO 17100-certified). The translation was carried out by one and edited by a second specialized translator. The final translation was reviewed by a group of experts based on existing guidelines for the cross-cultural adaptation of self-report measures (e.g.,^61^). Furthermore, technology commitment was assessed using the 12-item Technology Commitment Short Scale (Kurzskala Technikbereitschaft^62^). This scale aims to measure the willingness to use technology based on three aspects, which include technology acceptance, technology competence, and technology control beliefs (sum scores range between 12 and 60, higher values reflect higher technology commitment). Both scales exhibit good psychometric properties,^60,62^ which is also reflected in good internal consistency in our sample (Table A1 in Appendix 2).

Finally, patient-reported provider characteristics were assessed, comprising the provider’s attitude towards and skills for using telemental health services. Using two separate items, our participants were asked whether they agree that their mental health provider has a positive and open attitude towards the services and has the necessary skills to use the services without any problems. The response format for both items was a 5-point Likert scale ranging from 1 = strongly disagree to 5 = strongly agree.

### Statistical analysis

We first analyzed key sample characteristics descriptively. Then we tested the association of the included determinants with the continuous dependent variables using multiple linear regressions. Model assumptions for linear regressions were tested in advance (e.g., absence of multicollinearity). In addition, robust standard errors were employed to account for heteroscedasticity. In addition, subgroup analyses were conducted for both outcomes, including separate analyses for different service types (video, telephone, asynchronous services) and the two most prevalent psychiatric diagnoses in Germany (anxiety and depression^63^). Missing data were only present in the household income variable (3.6% missing). Consequently, listwise deletion was applied to deal with missing data. An alpha level of *p*<.05 was considered for statistical significance. Version 18.0 of Stata (StataCorp) was used for all of the statistical analyses, and the “alpha” command and “omegacoef” tool^64^ were used to calculate Cronbach’s alpha and McDonald’s omega, respectively, for the included scales.

## Results

### Sample characteristics

The descriptive characteristics of the examined sample are displayed in Table 1. Overall, 61% (586/961) of the sample were female and 39% (375/961) male. The sample consisted of individuals from the general adult population (18-74 years) with a mean age of 43 years (SD=11.2). The mean probability of reusing telemental health services in our sample was 65.8% (SD=30.6). On average, the probability of recommending telemental health services was 64.3% (SD=28.3).

**Table 1.** Descriptive sample characteristics (n = 961).

Variables	Values
*Patient intention to reuse and recommend telemental health services*	
Probability of reusing (range 0-100%), mean (SD)	65.8 (30.6)
Probability of recommending (range 0-100%), mean (SD)	64.3 (28.3)
*Socioeconomic factors*	
Sex, n (%)	
Male	375 (39.0)
Female	586 (61.0)
Age (in years), mean (SD)	43.0 (11.2)
Educational level^a^ (ISCED-97), n (%)	
Low educational level (ISCED 0-2)	103 (10.7)
Medium educational level (ISCED 3-4)	445 (46.3)
High educational level (ISCED 5-6)	413 (43.0)
Employment status, n (%)	
Unemployed	252 (26.2)
Full-time employed	444 (46.2)
Part-time employed	194 (20.2)
Other	71 (7.4)
Monthly household net income (€)^b^, n (%)	
Low income (0 to under 2000)	312 (33.7)
Medium income (2000 to under 3500)	315 (34.0)
High income (3500 or higher)	299 (32.3)
Migration background, n (%)	
Yes	146 (15.2)
No	815 (84.8)
Area lived in, n (%)	
Urban	513 (53.4)
Mostly urban	319 (33.2)
Rural	129 (13.4)
Relationship status, n (%)	
In a relationship	608 (63.3)
No partner	353 (36.7)
*Access factors*	
Insurance status, n (%)	
Statutory health insurance	904 (94.1)
Private health insurance	57 (5.9)
Quality of internet connection, n (%)	
Fast and stable	676 (70.3)
Fast, but not stable	130 (13.5)
Stable, but not fast	100 (10.4)
Neither fast nor stable or no internet connection at home	55 (5.7)
*Health factors*	
Depressive symptoms^c^ (range 0‐27), mean (SD)	12.7 (6.4)
Anxiety symptoms^d^ (range 0‐21), mean (SD)	10.3 (5.3)
Presence of at least 1 chronic physical illness, n (%)	
Yes	436 (45.4)
No	525 (54.6)
Self-rated health (range 1‐5), mean (SD)	3.0 (0.9)
*Psychosocial factors*	
Loneliness^e^ (mean score range 1‐4), mean (SD)	2.5 (0.6)
Perceived social support by family and friends^f^ (range 0‐30), mean (SD)	12.4 (5.7)
Self-efficacy^g^ (mean score range 1‐5), mean (SD)	3.4 (0.9)
Life satisfaction^h^ (range 5‐35), mean (SD)	3.6 (1.5)
*Personality* ^i^	
Conscientiousness (range 3‐21), mean (SD)	15.8 (3.4)
Extraversion (range 3‐21), mean (SD)	12.6 (3.9)
Agreeableness (range 3‐21), mean (SD)	15.2 (3.1)
Openness (range 3‐21), mean (SD)	14.5 (3.8)
Neuroticism (range 3‐21), mean (SD)	14.9 (3.6)
*Patient preferences*	
Attitude towards telemental health services^j^ (range 14‐70), mean (SD)	46.6 (10.1)
Technology commitment^k^ (range 12‐60), mean (SD)	42.2 (7.9)
*Provider characteristics*	
Provider attitude towards telemental health services (range 1‐5), mean (SD)	3.7 (1.1)
Provider skills for using telemental health services (range 1‐5), mean (SD)	3.9 (1.0)

*Notes*.^a^The educational level was measured using the International Standard Classification of Education 1997 (ISCED-97). Higher values indicate a higher educational level.

^b^Conversion rate: US $1=€0.924 in 2023.

^c^Depressive symptoms were measured using the Patient Health Questionnaire-9 (PHQ-9). Higher values indicate higher depressive symptoms.

^d^Anxiety symptoms were measured using the Generalized Anxiety Disorder Scale-7 (GAD-7). Higher values indicate higher anxiety symptoms.

^e^Loneliness was measured using the De Jong Gierveld Loneliness Scale. Higher values indicate higher loneliness.

^f^Perceived social support by family and friends was measured using the Lubben Social Network Scale-6 (LSNS-6). Higher values indicate higher perceived social support by family and friends.

^g^General self-efficacy was measured using the Short Scale for Measuring General Self-efficacy Beliefs (Allgemeine Selbstwirksamkeit Kurzskala [ASKU]). Higher values indicate higher self-efficacy.

^h^Life satisfaction was measured using the Satisfaction with Life Scale (SWLS). Higher values indicate higher life satisfaction.

^i^Personality was measured using the Big Five Inventory–Socio-Economic Panel (BFI-S). Higher values indicate higher levels of the different personality traits.

^j^Attitude towards telemental health services was measured using the Unified Theory of Acceptance and Use of Technology-Patient Version (UTAUT-P). Higher values indicate more positive attitudes towards telemental health services.

^k^Technology commitment was measured using the Technology Commitment Short Scale (Kurzskala Technikbereitschaft). Higher values indicate higher technology commitment.

### Regression analyses

Multiple linear regressions were computed to test for associations between the included determinants and the probability of reusing or recommending telemental health services (see Table A1 in Appendix 3 for detailed results). Statistically significant positive (or negative) coefficients indicate higher (or lower) intentions to reuse or recommend among patients, respectively. For example, a coefficient of *β=*0.50 means that a one-unit increase in the determinant is associated with a 0.50 percentage point increase (or decrease, if negative) in the intention to reuse or recommend, holding all other variables constant.

#### Determinants of the intention to reuse telemental health services

**Figure 1.**
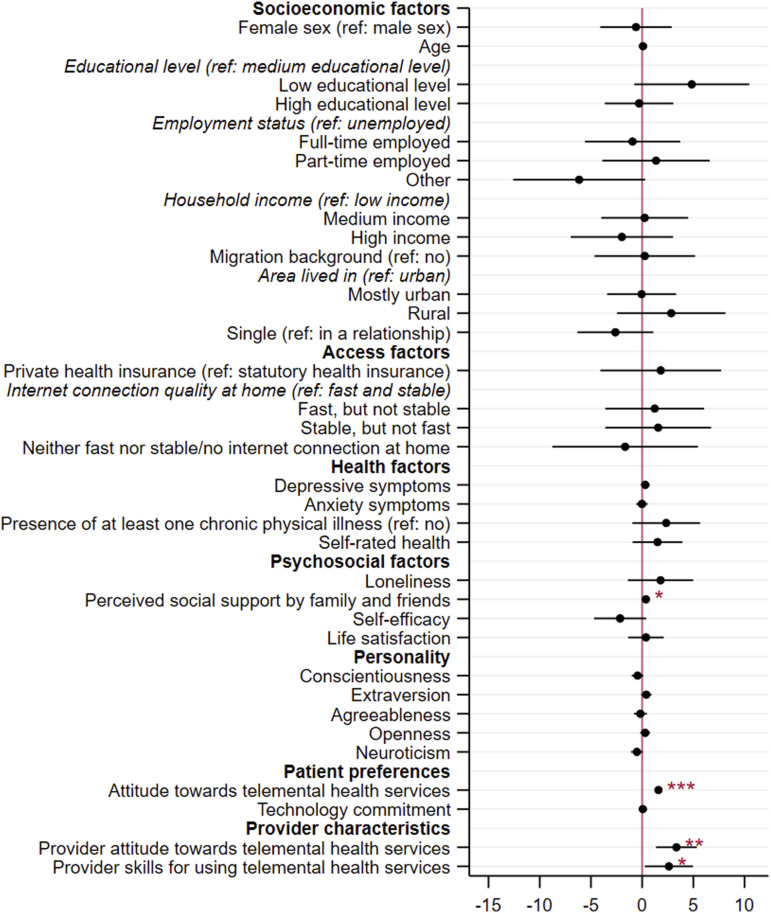
Results of multiple linear regression for determinants of reusing telemental health services (n = 926). *Note.* Beta coefficients with 95% CIs are presented. Ref=reference category. *** *p*<.001, ** *p*<.01, * *p*<.05.

Greater perceived social support (*β*=0.37, *p*=.039) and a more positive patient attitude towards telemental health services (*β*=1.60, *p*<.001) were positively associated with higher intention of reusing the services (Figure 1). In addition, provider characteristics, including a more positive patient-reported provider attitude towards (*β*=3.35, *p*=.001) and better provider skills for using telemental health services (*β*=2.61, *p*=.029), were positively associated with greater patient intention to reuse the services. Furthermore, subgroup analyses for service type and psychiatric diagnosis were conducted for this outcome. The subgroup analyses largely supported the observed significant relationships despite revealing some additional associations (see Table A2-A3 in Appendix 3 for more detailed results).

#### Determinants of the intention to recommend telemental health services

**Figure 2.**
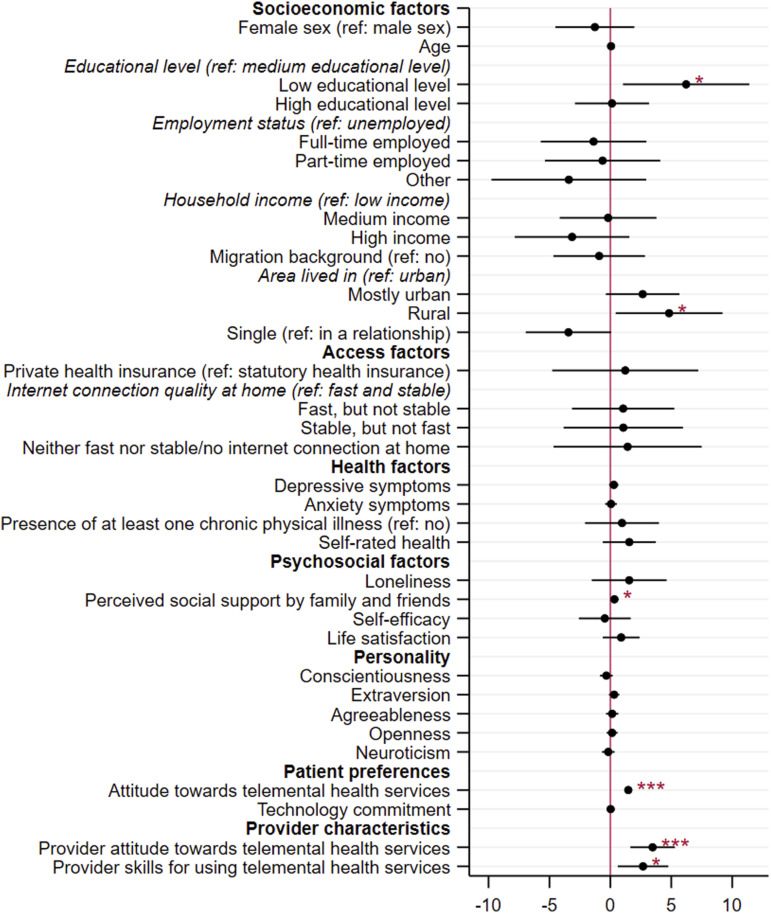
Results of multiple linear regression for determinants of recommending telemental health services (n = 926). *Note.* Beta coefficients with 95% CIs are presented. Ref=reference category. *** *p*<.001, ** *p*<.01, * *p*<.05.

Having a low educational level (*β*=6.22, *p*=.019), living in a rural area (*β*=4.82, *p*=.031), greater perceived social support (*β*=0.33, *p*=.044), and a more positive attitude towards telemental health services (*β*=1.47, *p*<.001) were found to be positively associated with a higher patient intention to recommend telemental health services (Figure 2). Also, a more positive patient-reported provider attitude (*β*=3.47, *p*<.001) and greater provider skills for using the services (*β*=2.68, *p*=.011) were positively associated with a higher patient intention to recommend telemental health services. The additional subgroup analyses mainly confirmed the observed significant associations and showed some additional relationships (see Table A4-A5 in Appendix 3 for more detailed results). However, perceived social support was not significantly associated with the likelihood of recommending telemental health services in any of the subgroup analyses (*p*>.05).

## Discussion

### Key findings

The objective of this study was to examine determinants of the intention to reuse and recommend telemental health services among mental health patients. We observed significant associations of perceived social support and the patients’ attitude towards the services with both outcomes. In addition, provider attitudes towards telemental health services and their skills in using them were both associated with the intention to reuse and recommend the services. Furthermore, a low educational level and living in a rural area were associated with the intention to recommend telemental health services. Access and health factors, as well as the patients’ personality, were not significantly related to the outcomes. Despite some additional relationships, subgroup analyses for different service types and psychiatric diagnoses largely supported these relationships. Nevertheless, a few contrasting results were examined, particularly for asynchronous services and the relationship of social support with the outcomes. Given that the existing literature offers little to no empirical observation of these associations, our study provides valuable and novel insights that can significantly advance understanding of sustained telemental health service use and recommendation behaviors. In this respect, our findings can serve as an important initial basis to guide future research.

### Relation to previous studies and implications

We found mostly non-significant associations between socioeconomic characteristics and both the intention to reuse and recommend telemental health services. Although female gender has been repeatedly linked to the continued use of telemental health services in previous international studies,^22,23^ our findings did not support this association. However, our findings align with international research concerning non-significant and mixed relationships of other socioeconomic determinants, including age, educational level, marital status, employment status, and ethnicity.^22^ Consequently, socioeconomic factors do not seem to impede the continued use of telemental health services and appear suitable for patients across all socioeconomic groups, at least within the German mental health care system. In terms of recommending telemental health services, there has been limited research to date, but one study on the willingness to recommend telehealth cancer genetic counseling also found no relationship with socioeconomic factors (including age, sex, and education).^65^ However, a low educational level and living in a rural area were associated with a greater likelihood of recommending telemental health services in our sample, indicating areas of application and potential to close health care gaps. Telemental health services might particularly improve access to care for patients with low education (e.g., easier scheduling of appointments, avoidance of stigmatization) and rural residence (e.g., travelling to appointments is not required, limited access to mental health care in local area), facilitating perceived value of and satisfaction with the services and thus recommendation likelihood.^66^ Furthermore, previous studies have also shown that individuals with lower education rely more on social resources when making health decisions^67,68^ and have higher trust in health advice from family and friends,^69^ which highlights the importance of recommendations in these groups.

Access and health determinants were not associated with the patients’ willingness to reuse and recommend telemental health services, which is in line with studies on patient use and satisfaction across different telemedicine services in German and international mental health patient samples (e.g.,^28,34,39^). This finding suggests that telemedicine is generally accessible and feasible for most patient groups, particularly within the German healthcare system. However, access barriers, such as technology access or technology infrastructure, should be carefully considered when examining different populations in future studies, as they may pose greater individual challenges to the use and continued use of telehealth services in other countries. For instance, a scoping review encompassing 395 studies observed that, compared to non-US and multicountry studies, US-based samples most frequently reported lack of access to technology as a barrier to telehealth use, underscoring regional disparities.^70^

Higher perceived social support was associated with both higher intentions to reuse and recommend telemental health services, which aligns with findings in the field of general telemedicine adoption.^71,72^ This could mean that social support by family and friends is related to the continued use of telemental health services. Family and friends may motivate better health behavior (e.g.,^73^), can facilitate trust in digital health services (e.g.,^74^), and might aid in using the services effectively (e.g., technical support^71^). Furthermore, patients with greater social support may depend less on in-person contacts with providers, which often provide valuable social interaction, thereby making them more open to using telemedicine services. Nevertheless, given the cross-sectional design of our study, the directionality of this relationship cannot be determined. While social support may encourage positive behavioral intentions, it is also possible that patients who have had positive experiences with the services are more likely to discuss these experiences with others, thereby reinforcing or increasing their perceived social support. Although perceived social support was significantly associated with the outcomes in the main analysis, no relationship was found in any of the subgroup analyses. This may be attributed to limited statistical power within the subgroups or might suggest that overall associations are not consistent across different patient groups or service types. Additional studies are needed to clarify this in the future. All of the other included psychosocial determinants were not associated with the outcomes. Existing research on psychosocial determinants is limited and yields mixed findings (e.g.,^22,28,34^), highlighting the general need for further investigation into their impact. Nonetheless, reuse and recommendation of telemental health services may be more strongly influenced by social support rather than individual psychosocial patient characteristics such as personality or self-efficacy, which may be more relevant to initial adoption of telemedicine services (e.g.,^75,76^).

While the patients’ individual level of technology commitment was not associated with the outcomes, more positive patient attitudes towards telemental health services were a key predictor of greater intention to reuse and recommend the services. This is supported by previous studies, which examined the relationship between individual attitudes or treatment expectancy and the use and continued use of telemental health services.^22,34^ This relationship underscores the crucial need to emphasize the benefits and effectiveness of remote mental health care, while also addressing prejudices among patients and the general population to foster its continuous and widespread use. Moreover, continuous targeted service improvements and innovations informed by individual user feedback and expectations are essential to support adherence to remote mental health treatment.^77^

The association of provider characteristics with the intention to reuse and recommend telemental health services was a central finding of our study. Provider attitudes and skills can positively influence patient satisfaction with the services,^39,78^ which could explain this relationship. Moreover, providers who are more open to and skilled in integrating telemental health into long-term treatments are likely better positioned to promote its sustained use. Therefore, more favorable provider attitudes towards the services and greater provider skills in using them can be critical to facilitate continuous use and recommendation of telemental health services. The recent literature suggests that mental health care providers generally exhibit positive attitudes towards telemental health services, but report concerns regarding effectiveness, technological issues, increased hassle, and impairment of the patient-provider relationship, which need to be resolved in the future.^79,80^ The strength of the patient-provider relationship may be a particularly critical factor in positively influencing patient engagement and intentions.^11,81^ Systematic reviews and meta-analyses indicate that the therapeutic alliance in telemental health care can be comparable to in-person services, with the most consistent evidence observed for synchronous modalities.^82–84^ Consequently, practical and research efforts should not only target patients to encourage the continuous and widespread use of telemental health services but also focus on supporting and training mental health providers in the effective delivery of telemental health care.

The subgroup analyses revealed that determinants may differ for different telemedicine modalities, consistent with existing research on determinants of patient use and satisfaction with telemedicine.^28,34,39,85^ While associations in the subgroup analyses for both examined synchronous services (video and telephone services) were quite similar to the main analyses, determinants for reusing and recommending asynchronous services showed greater differences. Asynchronous services may be more diverse and context-dependent. Consequently, efforts to facilitate reuse and recommendations of asynchronous services may require more specific tailoring, whereas approaches for synchronous services might be more broadly applicable.

### Strengths and limitations

We considered a large, representative sample of mental health patients to examine determinants of patient intention to reuse and recommend telemental health services. A wide range of determinants was included, guided by theoretical models and considerations, which have rarely or never been examined in previous studies. The determinants were measured using validated and established tools. Moreover, we aimed to represent most services of the telemedicine landscape, considering video, telephone, and asynchronous services individually. To the best of our knowledge, this is the first study to examine determinants of the intention to recommend telemental health services.

However, our study has some limitations. We used cross-sectional data. Based on this, we cannot observe causal relationships or longitudinal use and recommendation developments. Future longitudinal studies are needed to explore this in more detail. Additionally, relying exclusively on self-reported, single-source data may pose limitations, and we cannot entirely rule out the potential influence of biases such as common method, recall, or social desirability bias. Study participants were recruited through an online panel provider, which may have introduced selection bias by favoring individuals with greater familiarity with online and digital services. This could have biased responses towards higher acceptance of telemental health services and limits the generalizability of the findings to populations with limited access or experience with online or digital services. Furthermore, provider characteristics were measured using patient-reported data, which may have caused some degree of bias. However, patients’ perceptions of their telemedicine providers are commonly measured using self-reports (e.g.,^86^) and deliver important insights into patient experiences and how they may affect their behavior. Nevertheless, future studies may include additional data from providers to make more reliable conclusions. In addition, our assessment was limited to the evaluation of the likelihood of patients reusing and recommending telemental health services. Therefore, we cannot make conclusions regarding actual reuse and recommendations. In addition, the assessment of patient intentions was limited to single-item slider scales, which may be less reliable and provide less robust validity than multi-item scales, although single-item measures can perform well for unidimensional constructs such as intentions to reuse or recommend health services.^87^ Finally, the identified determinants may not be generalizable to other countries due to differences in telemedicine access and regulations, which warrants further investigation in future studies.

## Conclusion

Fostering the reuse and recommendation of telemental health services is vital for their widespread adoption and long-term success. Our findings firstly suggest that patients with greater social support and more positive attitudes towards the services are more likely to reuse and recommend them, indicating that they may be particularly suited for long-term telemental health treatment. In practice, they could serve as initial target groups for telemental health care initiatives, with additional efforts should be made to address the needs of individuals with less social support or more negative perceptions. Therefore, ongoing efforts to monitor and reinforce social connections, along with targeted educational campaigns to increase awareness and acceptance of telemental health services among mental health patients, can serve as crucial tools to enhance the long-term effectiveness of telemental health services.

Second, the recommendation of telemental health services may be particularly prevalent among patient groups with low educational levels and those residing in rural areas. For these populations, personal recommendations and targeted outreach may help to overcome barriers such as limited health literacy or access issues, ultimately bridging gaps in mental health care delivery. Implementation of community-based support groups tailored to rural populations and individuals with lower education could be considered by practitioners and policymakers to promote sustained engagement telemental health services.

Finally, mental health care providers appear as key advocates in sustaining the continuous use and recommendation of the services. Their active involvement and endorsement may help to build credibility and trust, address concerns, and foster patient confidence in using the services. Thus, efforts to cultivate positive attitudes and strengthen skills related to telemedicine among mental health care providers should be a central priority in both research and clinical practice.

## Supplemental material

Supplemental material - But would you use it again? Determinants of patient intention to reuse and recommend telemental health services: Representative cross-sectional survey from Germany Supplemental material for But would you use it again? Determinants of patient intention to reuse and recommend telemental health services: Representative cross-sectional survey from Germany by Ariana Neumann, Hans-Helmut König and André Hajek in Digital Health.

Supplemental material - But would you use it again? Determinants of patient intention to reuse and recommend telemental health services: Representative cross-sectional survey from Germany Supplemental material for But would you use it again? Determinants of patient intention to reuse and recommend telemental health services: Representative cross-sectional survey from Germany by Ariana Neumann, Hans-Helmut König and André Hajek in Digital Health.

Supplemental material - But would you use it again? Determinants of patient intention to reuse and recommend telemental health services: Representative cross-sectional survey from Germany Supplemental material for But would you use it again? Determinants of patient intention to reuse and recommend telemental health services: Representative cross-sectional survey from Germany by Ariana Neumann, Hans-Helmut König and André Hajek in Digital Health.

Supplemental material - But would you use it again? Determinants of patient intention to reuse and recommend telemental health services: Representative cross-sectional survey from Germany Supplemental material for But would you use it again? Determinants of patient intention to reuse and recommend telemental health services: Representative cross-sectional survey from Germany by Ariana Neumann, Hans-Helmut König and André Hajek in Digital Health.

## Data Availability

The data that support the findings of this study are available from the corresponding author upon reasonable request.*
