# Supplementary material for: But would you use it again? Determinants of patient intention to reuse and recommend telemental health services: Representative cross-sectional survey from Germany
Source: Digit Health. 2026 Jul 15;12:20552076261450732. doi: 10.1177/20552076261450732 (PMC13373393; doi:10.1177/20552076261450732)
Supplement: Supplemental material - But would you use it again? Determinants of patient intention to reuse and recommend telemental health services: Representative cross-sectional survey from Germany [file sj-pdf-2-dhj-10.1177_20552076261450732.pdf]

Appendix 2

Table A1. Internal Consistency of the Included Scales.

Scales	Cronbach's Alpha	McDonald's Omega
Patient Health Questionnaire-9	0.89	0.89
Generalized Anxiety Disorder Scale-7	0.89	0.90
De Jong Gierveld Loneliness Scale	0.78	0.77
Lubben Social Network Scale	0.86	0.84
Short Scale for Measuring General Self-Efficacy Beliefs	0.88	0.88
Satisfaction with Life Scale	0.92	0.92
Big Five Inventory–Socio-Economic Panel		
Conscientiousness	0.63	0.69
Extraversion	0.72	0.74
Agreeableness	0.49	0.56
Openness	0.70	0.70
Neuroticism	0.63	0.66
Unified Theory of Acceptance and Use of Technology-Patient Version Questionnaire	0.86	0.85
Technology Commitment Short Scale	0.81	0.77
