# Supplementary material for: But would you use it again? Determinants of patient intention to reuse and recommend telemental health services: Representative cross-sectional survey from Germany
Source: Digit Health. 2026 Jul 15;12:20552076261450732. doi: 10.1177/20552076261450732 (PMC13373393; doi:10.1177/20552076261450732)
Supplement: Supplemental material - But would you use it again? Determinants of patient intention to reuse and recommend telemental health services: Representative cross-sectional survey from Germany [file sj-pdf-3-dhj-10.1177_20552076261450732.pdf]

Appendix 3

Table A1. Results of multiple linear regressions for determinants of reusing and recommending telemental health services.

Variables	Intention to Reuse	Intention to Recommend
Socioeconomic factors		
Female sex (ref: male sex)	-0.60	-1.27
	(1.78)	(1.65)
Age	0.08	0.06
	(0.09)	(0.08)
Educational level (ref: medium educational level)		
Low educational level	4.85+	6.22*
	(2.86)	(2.64)
High educational level	-0.30	0.13
	(1.71)	(1.55)
Employment status (ref: unemployed)		
Full-time employed	-0.93	-1.38
	(2.37)	(2.21)
Part-time employed	1.36	-0.64
	(2.67)	(2.41)
Other	-6.15+	-3.41
	(3.29)	(3.24)
Household income (ref: low income)		
Medium income	0.25	-0.19
	(2.16)	(2.02)
High income	-1.98	-3.14
	(2.55)	(2.40)
Migration background (ref: no)	0.26	-0.92
	(2.50)	(1.91)
Area lived in (ref: urban)		
Mostly urban	-0.05	2.65+
	(1.72)	(1.54)
Rural	2.84	4.82*
	(2.69)	(2.23)
Single (ref: in a relationship)	-2.61	-3.44+
	(1.89)	(1.79)
Access factors		
Private health insurance (ref: statutory health insurance)	1.82	1.22
	(3.01)	(3.05)
Internet connection quality at home (ref: fast and stable)		
Fast, but not stable	1.23	1.05
	(2.46)	(2.14)
Stable, but not fast	1.57	1.06
	(2.63)	(2.50)
Neither fast nor stable/no internet connection at home	-1.66	1.42
	(3.62)	(3.10)
Health factors		
Depressive symptoms	0.31	0.28
	(0.22)	(0.19)
Anxiety symptoms	-0.02	0.05

	(0.28)	(0.25)
Presence of at least one chronic physical illness (ref: no)	2.35	0.96
	(1.68)	(1.54)
Self-rated health	1.50	1.55
	(1.24)	(1.11)
Psychosocial factors		
Loneliness	1.80	1.55
	(1.63)	(1.56)
Perceived social support by family and friends	0.37*	0.33*
	(0.18)	(0.16)
Self-efficacy	-2.15+	-0.46
	(1.30)	(1.09)
Life satisfaction	0.37	0.88
	(0.89)	(0.77)
Personality		
Conscientiousness	-0.45	-0.33
	(0.29)	(0.26)
Extraversion	0.41	0.31
	(0.25)	(0.22)
Agreeableness	-0.17	0.15
	(0.32)	(0.25)
Openness	0.30	0.14
	(0.25)	(0.23)
Neuroticism	-0.51+	-0.17
	(0.29)	(0.26)
Patient preferences		
Attitude towards telemental health services	1.60***	1.47***
	(0.11)	(0.09)
Technology commitment	0.07	0.02
	(0.13)	(0.11)
Provider characteristics		
Provider attitude towards telemental health services	3.35**	3.47***
	(1.02)	(0.93)
Provider skills for using telemental health services	2.61*	2.68*
	(1.20)	(1.06)
Constant	-40.65***	-48.37***
	(11.30)	(10.42)
Observations	926	926
R-squared	0.436	0.461
Adjusted R-squared	0.415	0.440
RMSE	23.23	21.02

Notes. Beta coefficients are reported. Robust standard errors in parentheses. Ref = reference category. *** p<0.001, ** p<0.01, * p<0.05, + p<0.10.

Table A2. Results of multiple linear regressions for determinants of reusing video, telephone, or asynchronous telemental health services.

Variables	Video	Telephone	Asynchronous
Socioeconomic factors			
Female sex (ref: male sex)	-2.69	-2.18	3.32
	(1.99)	(2.40)	(2.75)
Age	0.05	-0.15	0.20
	(0.10)	(0.12)	(0.16)
Educational level (ref: medium educational level)			
Low educational level	3.32	8.27*	-0.75
	(3.29)	(3.91)	(6.44)
High educational level	-1.51	-2.83	-2.35
	(1.97)	(2.40)	(3.16)
Employment status (ref: unemployed)			
Full-time employed	-0.58	-4.93	-3.85
	(2.75)	(3.25)	(6.17)
Part-time employed	4.55	-1.58	2.32
	(3.02)	(3.53)	(6.76)
Other	0.73	-17.07***	-0.59
	(3.77)	(4.88)	(8.84)
Household income (ref: low income)			
Medium income	1.00	-2.15	9.13*
	(2.59)	(3.27)	(4.57)
High income	-1.63	-4.35	2.48
	(2.85)	(3.78)	(5.19)
Migration background (ref: no)	-0.39	0.88	3.28
	(2.65)	(3.36)	(3.95)
Area lived in (ref: urban)			
Mostly urban	-1.78	0.66	0.25
	(2.06)	(2.47)	(3.34)
Rural	-0.16	3.98	-1.17
	(2.91)	(3.35)	(5.25)
Single (ref: in a relationship)	-1.92	-4.85+	-1.43
	(2.16)	(2.94)	(3.60)
Access factors			
Private health insurance (ref: statutory health insurance)	4.40	6.11	0.45
	(3.24)	(4.32)	(4.17)
Internet connection quality at home (ref: fast and stable)			
Fast, but not stable	4.48+	-2.22	-1.65
	(2.55)	(3.27)	(4.27)
Stable, but not fast	0.18	-3.25	4.57
	(3.24)	(3.66)	(6.07)
Neither fast nor stable/no internet connection at home	-0.00	-3.67	4.43
	(5.90)	(5.38)	(8.96)
Health factors			
Depressive symptoms	0.56*	0.09	0.06
	(0.25)	(0.34)	(0.42)
Anxiety symptoms	-0.01	-0.07	0.25
	(0.33)	(0.46)	(0.56)
Presence of at least one chronic physical illness (ref: no)	1.66	0.92	0.45

	(1.97)	(2.39)	(3.37)
Self-rated health	1.64	-0.32	0.31
	(1.35)	(1.76)	(2.44)
Psychosocial factors			
Loneliness	0.51	1.99	2.81
	(1.76)	(2.41)	(3.30)
Perceived social support by family and friends	0.39+	0.07	0.27
	(0.21)	(0.24)	(0.28)
Self-efficacy	-1.18	-3.54+	-3.58
	(1.52)	(2.00)	(2.37)
Life satisfaction	0.17	1.19	5.49***
	(0.95)	(1.27)	(1.59)
Personality			
Conscientiousness	-0.80*	0.05	-0.09
	(0.32)	(0.46)	(0.56)
Extraversion	0.46	0.54	-0.45
	(0.29)	(0.36)	(0.50)
Agreeableness	-0.37	-0.27	-0.15
	(0.33)	(0.53)	(0.56)
Openness	-0.16	0.27	0.07
	(0.26)	(0.37)	(0.54)
Neuroticism	-0.85*	-0.83+	-0.29
	(0.33)	(0.46)	(0.51)
Patient preferences			
Attitude towards telemental health services	1.66***	1.51***	1.43***
	(0.14)	(0.16)	(0.21)
Technology commitment	0.07	-0.31+	0.32
	(0.15)	(0.18)	(0.28)
Provider characteristics			
Provider attitude towards telemental health services	2.93*	1.65	2.61
	(1.26)	(1.57)	(2.12)
Provider skills for using telemental health services	3.09*	4.69*	3.81+
	(1.51)	(1.91)	(2.14)
Constant	-25.06 +	9.18	-59.84**
	(13.45)	(16.79)	(19.54)
Observations	483	428	257
R-squared	0.510	0.424	0.516
Adjusted R-squared	0.473	0.374	0.441
RMSE	19.04	21.98	20.95

Notes. Beta coefficients are reported. Robust standard errors in parentheses. Ref = reference category. *** p<0.001, ** p<0.01, * p<0.05, + p<0.10.

Table A3. Results of multiple linear regressions for determinants of reusing telemental health services in patients with depression and anxiety.

Variables	Anxiety	Depression
Socioeconomic factors		
Female sex (ref: male sex)	-4.75+	-1.17
	(2.42)	(2.31)
Age	0.01	0.10
	(0.11)	(0.10)
Educational level (ref: medium educational level)		
Low educational level	6.56*	6.23+
	(3.07)	(3.56)
High educational level	2.89	1.80
	(2.52)	(2.11)
Employment status (ref: unemployed)		
Full-time employed	-2.93	-2.75
	(2.88)	(2.78)
Part-time employed	-1.43	0.40
	(3.33)	(3.14)
Other	-6.55	-6.52+
	(4.15)	(3.71)
Household income (ref: low income)		
Medium income	-1.94	0.23
	(2.96)	(2.65)
High income	-1.73	-0.57
	(3.48)	(3.17)
Migration background (ref: no)	0.71	-0.82
	(2.92)	(3.09)
Area lived in (ref: urban)		
Mostly urban	1.13	-1.22
	(2.24)	(2.08)
Rural	1.68	0.01
	(3.87)	(3.38)
Single (ref: in a relationship)	-2.50	-1.32
	(2.57)	(2.29)
Access factors		
Private health insurance (ref: statutory health insurance)	-0.92	2.30
	(3.86)	(3.61)
Internet connection quality at home (ref: fast and stable)		
Fast, but not stable	-1.18	2.33
	(3.34)	(2.88)
Stable, but not fast	-0.65	4.78
	(3.11)	(3.72)
Neither fast nor stable/no internet connection at home	-1.35	1.97
	(5.07)	(3.79)
Health factors		
Depressive symptoms	0.30	0.51+
	(0.29)	(0.27)
Anxiety symptoms	-0.17	-0.46
	(0.37)	(0.33)
Presence of at least one chronic physical illness (ref: no)	-0.12	4.72*
	(2.28)	(2.12)

Self-rated health	0.43	2.49
	(1.57)	(1.56)
Psychosocial factors		
Loneliness	1.06	1.99
	(2.15)	(2.10)
Perceived social support by family and friends	-0.15	0.46+
	(0.24)	(0.24)
Self-efficacy	0.58	-1.18
	(1.69)	(1.51)
Life satisfaction	1.20	1.15
	(1.05)	(1.10)
Personality		
Conscientiousness	-0.85*	-0.64+
	(0.40)	(0.35)
Extraversion	0.45	0.15
	(0.30)	(0.30)
Agreeableness	-0.24	-0.27
	(0.40)	(0.39)
Openness	0.11	0.33
	(0.33)	(0.29)
Neuroticism	-0.33	-0.16
	(0.40)	(0.37)
Patient preferences		
Attitude towards telemental health services	1.71***	1.74***
	(0.15)	(0.12)
Technology commitment	-0.18	-0.00
	(0.17)	(0.15)
Provider characteristics		
Provider attitude towards telemental health services	2.66+	2.18+
	(1.49)	(1.28)
Provider skills for using telemental health services	3.33+	3.41*
	(1.74)	(1.42)
Constant	-18.63	-51.36***
	(15.18)	(15.18)
Observations	489	626
R-squared	0.466	0.464
Adjusted R-squared	0.426	0.433
RMSE	22.39	23.57

Notes. Beta coefficients are reported. Robust standard errors in parentheses. Ref = reference category. *** p<0.001, ** p<0.01, * p<0.05, + p<0.10.

Table A4. Results of multiple linear regressions for determinants of recommending video, telephone, or asynchronous telemental health services.

Variables	Video	Telephone	Asynchronous
Socioeconomic factors			
Female sex (ref: male sex)	-2.96	-3.41	3.14
	(1.87)	(2.10)	(2.80)
Age	-0.09	-0.11	0.21
	(0.09)	(0.10)	(0.14)
Educational level (ref: medium educational level)			
Low educational level	5.36+	9.37**	-0.19
	(3.03)	(3.42)	(5.62)
High educational level	-1.15	-0.03	-1.82
	(1.76)	(2.12)	(2.90)
Employment status (ref: unemployed)			
Full-time employed	-0.26	-4.51	-2.19
	(2.57)	(2.84)	(5.44)
Part-time employed	3.01	-4.59	4.42
	(2.74)	(3.18)	(5.69)
Other	5.91+	-11.48*	-0.30
	(3.11)	(4.54)	(8.16)
Household income (ref: low income)			
Medium income	-0.92	2.32	3.17
	(2.35)	(2.80)	(4.12)
High income	-4.62+	-0.45	-0.46
	(2.56)	(3.44)	(4.66)
Migration background (ref: no)	3.65	1.84	0.36
	(2.25)	(2.51)	(3.34)
Area lived in (ref: urban)			
Mostly urban	-0.93	3.90+	4.44
	(1.93)	(2.20)	(3.11)
Rural	3.20	8.30**	-0.39
	(2.46)	(2.62)	(3.79)
Single (ref: in a relationship)	-1.76	-3.07	-3.43
	(1.93)	(2.57)	(3.47)
Access factors			
Private health insurance (ref: statutory health insurance)	3.77	5.35	3.77
	(3.47)	(4.20)	(3.23)
Internet connection quality at home (ref: fast and stable)			
Fast, but not stable	0.79	0.24	2.58
	(2.31)	(3.00)	(3.27)
Stable, but not fast	-1.55	-1.46	2.06
	(2.96)	(3.51)	(5.81)
Neither fast nor stable/no internet connection at home	5.26	-3.45	1.97
	(3.42)	(3.71)	(8.04)
Health factors			
Depressive symptoms	0.31	0.05	0.10
	(0.24)	(0.28)	(0.37)
Anxiety symptoms	0.18	0.19	0.10
	(0.30)	(0.39)	(0.48)
Presence of at least one chronic physical illness (ref: no)	1.62	-1.02	1.33
	(1.79)	(2.11)	(2.91)

Self-rated health	-0.10	0.05	0.61
	(1.22)	(1.52)	(1.93)
Psychosocial factors			
Loneliness	2.82	1.44	1.53
	(1.72)	(2.15)	(2.93)
Perceived social support by family and friends	0.34+	0.14	0.18
	(0.20)	(0.21)	(0.27)
Self-efficacy	-0.64	-1.56	-3.09
	(1.22)	(1.45)	(2.04)
Life satisfaction	2.23*	1.00	4.07**
	(0.91)	(0.94)	(1.56)
Personality			
Conscientiousness	-0.76**	0.04	0.39
	(0.29)	(0.35)	(0.54)
Extraversion	0.13	0.56+	-0.31
	(0.24)	(0.31)	(0.43)
Agreeableness	0.19	0.27	-0.66
	(0.32)	(0.39)	(0.46)
Openness	0.14	-0.18	0.47
	(0.26)	(0.32)	(0.45)
Neuroticism	-0.90**	-0.23	-0.64
	(0.31)	(0.40)	(0.47)
Patient preferences			
Attitude towards telemental health services	1.78***	1.39***	1.41***
	(0.12)	(0.13)	(0.18)
Technology commitment	-0.01	-0.25+	0.06
	(0.13)	(0.15)	(0.21)
Provider characteristics			
Provider attitude towards telemental health services	2.40*	3.01*	2.41
	(1.11)	(1.27)	(1.74)
Provider skills for using telemental health services	0.63	4.66**	3.22+
	(1.35)	(1.59)	(1.70)
Constant	-28.00*	-21.93	-39.32*
	(12.76)	(14.63)	(19.39)
Observations	483	428	257
R-squared	0.554	0.481	0.534
Adjusted R-squared	0.520	0.436	0.463
RMSE	17.36	19.23	18.66

Notes. Beta coefficients are reported. Robust standard errors in parentheses. Ref = reference category. *** p<0.001, ** p<0.01, * p<0.05, + p<0.10.

Table A5. Results of multiple linear regressions for determinants of recommending telemental health services in patients with depression and anxiety.

Variables	Anxiety	Depression
Socioeconomic factors		
Female sex (ref: male sex)	-4.08+	-1.47
	(2.19)	(2.16)
Age	-0.02	0.10
	(0.10)	(0.10)
Educational level (ref: medium educational level)		
Low educational level	6.44*	3.93
	(2.81)	(3.38)
High educational level	1.36	0.65
	(2.14)	(1.87)
Employment status (ref: unemployed)		
Full-time employed	-2.43	-1.72
	(2.67)	(2.60)
Part-time employed	0.65	0.40
	(2.75)	(2.77)
Other	-6.07	-0.55
	(4.00)	(3.70)
Household income (ref: low income)		
Medium income	1.09	1.44
	(2.55)	(2.46)
High income	-0.22	-3.55
	(3.29)	(3.00)
Migration background (ref: no)	-0.81	-1.57
	(2.30)	(2.52)
Area lived in (ref: urban)		
Mostly urban	3.84*	1.55
	(1.93)	(1.86)
Rural	3.54	3.33
	(3.01)	(2.84)
Single (ref: in a relationship)	-1.46	-2.09
	(2.18)	(2.16)
Access factors		
Private health insurance (ref: statutory health insurance)	4.15	0.26
	(3.72)	(4.02)
Internet connection quality at home (ref: fast and stable)		
Fast, but not stable	1.05	1.60
	(3.08)	(2.75)
Stable, but not fast	-3.27	0.83
	(2.80)	(3.39)
Neither fast nor stable/no internet connection at home	-2.20	5.32
	(4.02)	(3.44)
Health factors		
Depressive symptoms	0.21	0.17
	(0.23)	(0.24)
Anxiety symptoms	0.13	-0.05
	(0.30)	(0.30)
Presence of at least one chronic physical illness (ref: no)	0.48	2.98
	(2.01)	(1.87)

Self-rated health	0.74	1.18
	(1.32)	(1.41)
Psychosocial factors		
Loneliness	0.29	0.78
	(1.87)	(2.05)
Perceived social support by family and friends	-0.17	0.39+
	(0.21)	(0.22)
Self-efficacy	0.66	0.03
	(1.37)	(1.27)
Life satisfaction	1.71+	1.23
	(0.95)	(1.00)
Personality		
Conscientiousness	-0.71*	-0.57+
	(0.34)	(0.34)
Extraversion	0.35	0.16
	(0.26)	(0.27)
Agreeableness	0.10	0.07
	(0.33)	(0.31)
Openness	0.00	0.16
	(0.27)	(0.26)
Neuroticism	-0.35	0.02
	(0.35)	(0.33)
Patient preferences		
Attitude towards telemental health services	1.67***	1.56***
	(0.12)	(0.11)
Technology commitment	-0.16	0.03
	(0.13)	(0.13)
Provider characteristics		
Provider attitude towards telemental health services	3.25*	3.77**
	(1.30)	(1.16)
Provider skills for using telemental health services	1.30	2.12
	(1.51)	(1.29)
Constant	-22.72+	-48.99***
	(13.15)	(13.77)
Observations	489	626
R-squared	0.526	0.483
Adjusted R-squared	0.490	0.453
RMSE	19.29	21.34

Notes. Beta coefficients are reported. Robust standard errors in parentheses. Ref = reference category. *** p<0.001, ** p<0.01, * p<0.05, + p<0.10.
