# Supplementary material for: But would you use it again? Determinants of patient intention to reuse and recommend telemental health services: Representative cross-sectional survey from Germany
Source: Digit Health. 2026 Jul 15;12:20552076261450732. doi: 10.1177/20552076261450732 (PMC13373393; doi:10.1177/20552076261450732)
Supplement: Supplemental material - But would you use it again? Determinants of patient intention to reuse and recommend telemental health services: Representative cross-sectional survey from Germany [file sj-pdf-4-dhj-10.1177_20552076261450732.pdf]

Appendix 4

Table A1. Included variables and their measurement.

Variables		Survey Items	Response
**Patient Intentions**	Intention to reuse telemental health services	Would you use telemental health services again in the future as part of your mental health treatment?	0-100% (probability slider)
	Intention to recommend telemental health services	Would you recommend telemental health services for mental health care?	0-100% (probability slider)
**Socioeconomic**	Sex	What is your sex?	Male, female
	Age	In which year were you born?	Year of birth
	Education	International Standard Classification of Education 97 (low, medium, high)	
	Employment status	What is your current employment situation?	Full-time employed, part-time employed, unemployed, other
	Household income	What is the average monthly net income of your household?	Less than 500 €, 500 € to under 1,000 €, 1,000 € to under 1,500 €, 1,500 € to under 2,000 €, 2,000 € to under 2,500 €, 2,500 € to under 3,000 €, 3,000 € to under 3,500 €, 3,500 € to under 4,000 €, 4,000 € to under 4,500 €, 4,500 € to under 5,000 €, 5,000 € to under 6,000 €, 6,000 € to under 8,000 €, 8,000 € or higher
	Migration background	Do you have a migrant background? A person has a migration background if they themselves or at least one of their parents were not born with German citizenship.	Yes, no
	Area lived in	What is the postcode of your current place of residence?	Categorization into urban, mostly urban, and rural according to postcode
	Relationship status	What is your current relationship status?	In a relationship, single
**Access**	Insurance type	What is your current insurance type?	Statutory health insurance, private health insurance
	Internet connection quality at home	Do you have a stable (uninterrupted) and fast (smooth, fast loading of content) Internet connection at home?	Yes, I have a fast and stable internet connection; My internet connection is fast, but not stable; My internet

			connection is stable, but not fast; No, my internet connection is neither fast nor stable/I do not have an internet connection at home
**Health**	Depression	9-item Patient Health Questionnaire-9 (PHQ-9), scores ranging from 0 to 27, higher values indicate more severe depressive symptoms	
	Anxiety	7-item Generalized Anxiety Disorder Scale-7 (GAD-7), scores ranging from 0 to 21, higher values indicate more severe anxiety symptoms	
	Physical illness	Do you have at least one chronic physical illness (e.g., diabetes, heart disease)?	Yes, no
	Self-rated health	How would you rate your current state of health?	Very bad, bad, average, good, very good
**Psychosocial**	Loneliness	6-item De Jong Gierveld Loneliness Scale, scores ranging from 1 to 4, higher values indicate higher levels of loneliness	
	Perceived social support	6-item Lubben Social Network Scale (LSNS-6), scores ranging from 1 to 30, higher values indicate greater perceived social support	
	General self-efficacy	3-item Short Scale for Measuring General Self-efficacy Beliefs ([Allgemeine Selbstwirksamkeit Kurzskala] ASKU), scores ranging from 1 to 5, higher values indicate greater self-efficacy	
	Life satisfaction	5-item German version of the Satisfaction with Life Scale (SWLS), scores ranging from 1 to 5, higher values indicate greater life satisfaction	
	Personality	15-item Big Five Inventory-SOEP (BFI-S), trait sum scores range between 3 and 21, higher values indicate higher levels of the different personality traits	
**Patient preferences**	Attitude towards telemental health services	14-item Unified Theory of Acceptance and Use of Technology-Patient version questionnaire (UTAUT-P), scores ranging from 14 to 70, higher values indicate more positive attitudes	
	Technology commitment	12-item Technology Commitment Short Scale (Kurzskala Technikbereitschaft), scores ranging from 12 to 60, higher values indicate higher technology commitment	
**Provider characteristics**	Attitude	Now think about your current psychotherapist/psychiatrist/doctor. To what extent do the following statements apply? If you are not currently undergoing mental health treatment, please think of your last psychotherapist/psychiatrist/doctor. My therapist has a positive and open attitude towards telemental health services (e.g., offers and advertises them).	Strongly disagree, disagree, neither, agree, strongly agree

	Skills	My therapist has the necessary skills to use telemental health services successfully and without problems (e.g., has technical skills and knowledge of the digital programs).	Strongly disagree, disagree, neither, agree, strongly agree
